# Supplementary material for: Cochaperones convey the energy of ATP hydrolysis for directional action of Hsp90
Source: Nat Commun. 2024 Jan 17;15:569. doi: 10.1038/s41467-024-44847-6 (PMC10794413; doi:10.1038/s41467-024-44847-6)
Supplement: Supplementary file 1 — Supplementary Information [file 41467_2024_44847_MOESM1_ESM.pdf]

## Cochaperones convey the energy of ATP hydrolysis for directional action of Hsp90

Leonie Vollmar[1, 2, 3], Julia Schimpf[1, 2, 3], Bianca Hermann[1], Thorsten Hugel[1,4]

[1]Institute of Physical Chemistry, University of Freiburg, Germany
[2]Spemann Graduate School of Biology and Medicine (SGBM), University of Freiburg
[3]These authors contributed equally: Leonie Vollmar, Julia Schimpf
[4]Signalling Research Centers BIOSS and CIBSS, University of Freiburg, Germany
Corresponding author: Thorsten Hugel, th@pc.uni-freiburg.de

## Supplementary Information

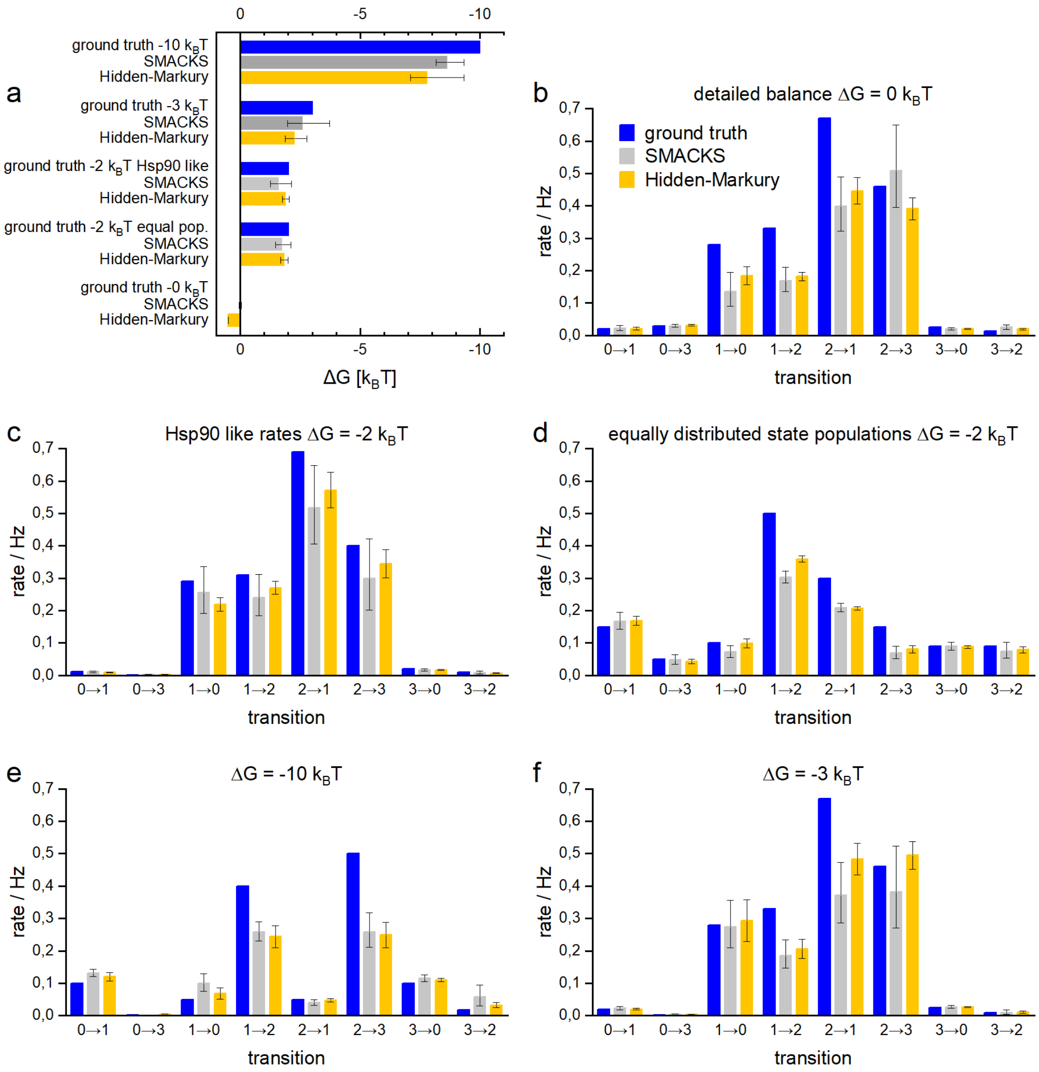


**Supplementary Fig. 1**: HMM analysis by SMACKS (grey) or Hidden-Markury (yellow) is able to retrieve kinetic rate constants and directionality of traces simulated with MASH-FRET. The underlying four-state model displays two low FRET states 0 and 1 ($E_{0 \text{ and } 1}$ = 0.1 ± 0.05) and two high FRET states 2 and 3 ($E_{2 \text{ and } 3}$ = 0.8 ± 0.05). Diagonal rates were set to zero. 200 traces with 200 frames at a frame rate of 2 Hz were simulated for each condition based on ground truth (blue). **a** Retrieved Gibb's free energy. Error bars calculated from 95% CIs of rate constants. **b** Model in detailed balance, i.e. ΔG = 0 $k_BT$. **c** Ground truth designed to have a directionality of ΔG = -2 $k_BT$ and rates very similar to those obtained from experiments with Hsp90, where a comparable directionality was measured. **d** Ground truth designed to have a directionality of ΔG = -2 $k_BT$ and equally distributed state populations: $P_0$ = 23%, $P_1$ = 20%, $P_2$ = 28%, $P_3$ = 29%. **e** Ground truth designed to have a larger directionality of ΔG = -10 $k_BT$. **f** Ground truth designed to have a directionality of ΔG = -3 $k_BT$ and Hsp90 like rates. Error bars for rates are 95% CIs. All values are given in Supplementary Data file 1: Source Data. Source data are provided as a Source Data file.

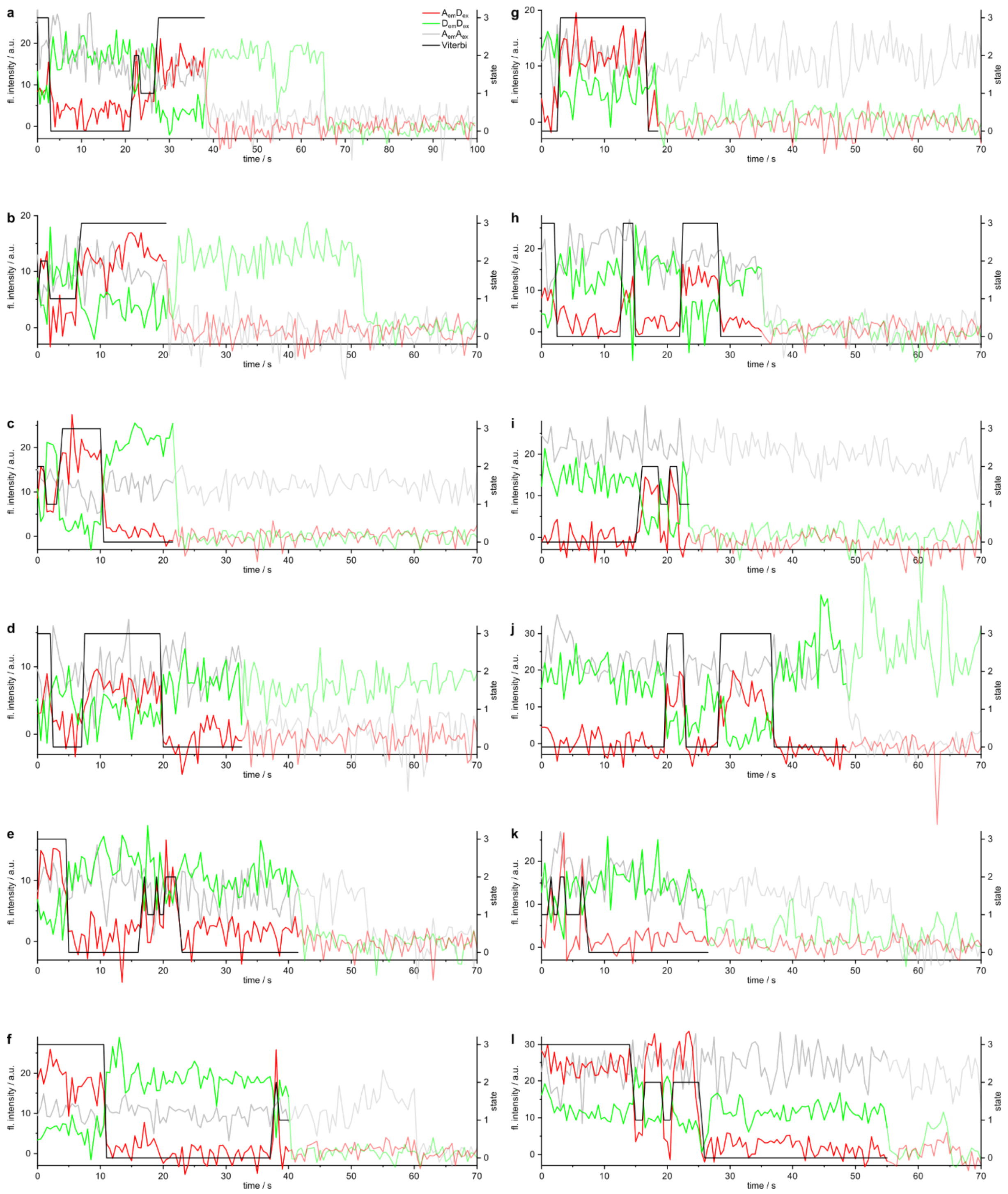


**Supplementary Fig. 2**: Exemplary single-molecule FRET traces showing Hsp90's opening and closing dynamics in presence of various cochaperones. Viterbi path (black) obtained for the part of the trace before photo bleaching (faded colors indicate at least one dye being bleached). FRET signal (acceptor emission after donor excitation) shown as red line, direct excitation of the donor shown in green, and of the acceptor in grey. Fluorescence intensity in arbitrary units. **a-f** $Cdc37_1$-$Hsp90_2$ + ATP + Ste11 + Aha1 + Sba1. **g-l** $Cdc37_1$-$Hsp90_2$ + ATP + Ste11. Source data are provided as a Source Data file.

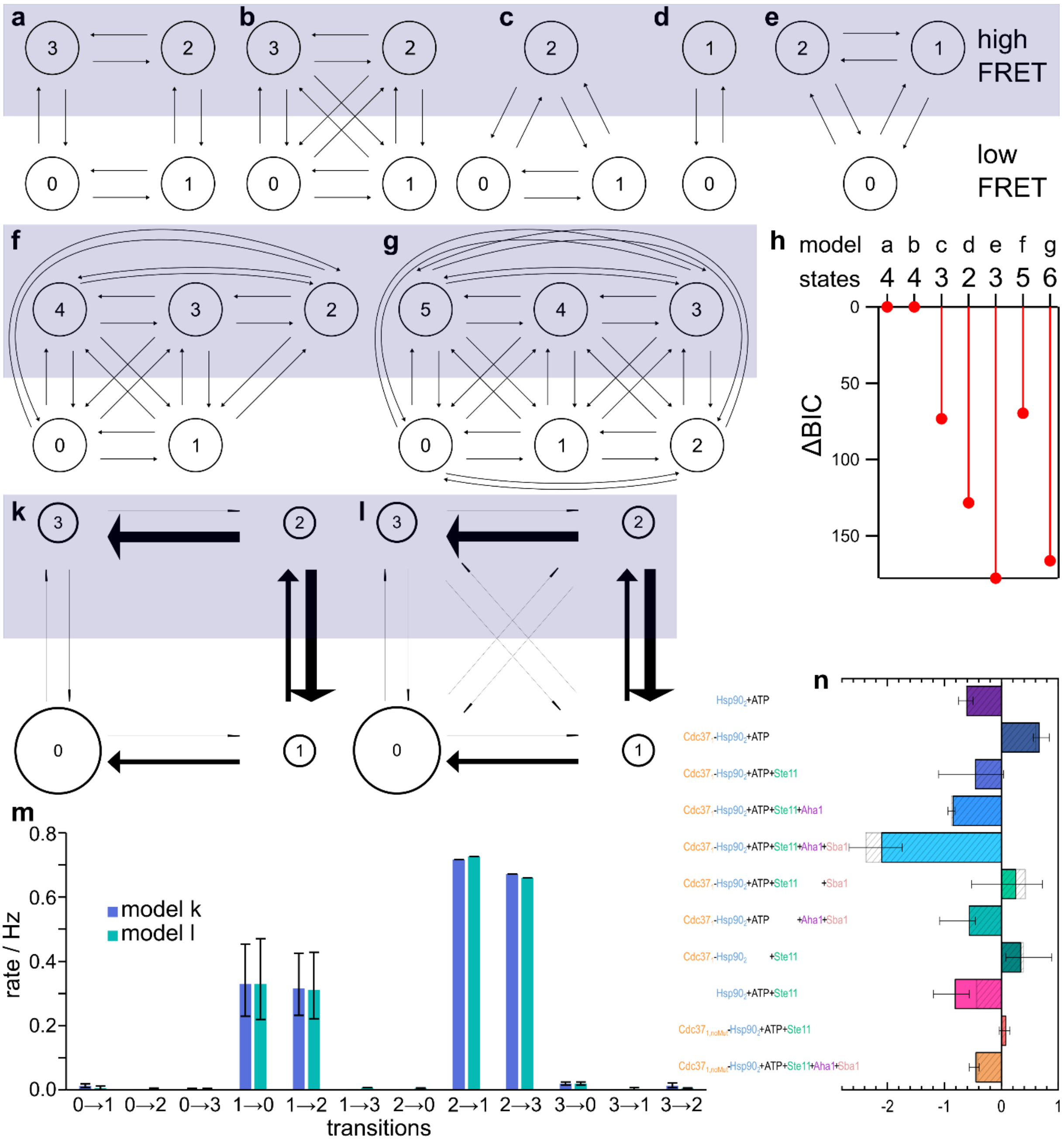


**Supplementary Fig. 3**: Selecting the best model describing Hsp90's conformational behaviour. The ensemble Hidden Markov modelling for all traces of one experiment (here: Cdc37$_1$-Hsp90$_2$ + ATP + Ste11 + Aha1 + Sba1, 291 traces) was carried out with models (**a-g**) of varying complexity and amounts of hidden states. High FRET states in grey. **h** A four state model best fits the data, having the lowest Bayesian Information Criterion (BIC). Model a and b have an identical BIC of 777690. **k,l** Representations of model a and b with circle sizes proportional to the state populations and arrow widths proportional to the transition rates. **m** Transition rates with 95 % confidence intervals of model k and l. Diagonal rates are close to zero. **n** Coloured bars: Calculated Gibb's free energies for each measured condition with model a. Grey hatched: calculated with model b. For both models, clear directionality can only be observed in presence of all three cochaperones (lightest blue). Together with the results in m, this substantiates our selection of model a. All calculations were done with SMACKS as described in the methods and Supplementary methods. Source data are provided as a Source Data file.

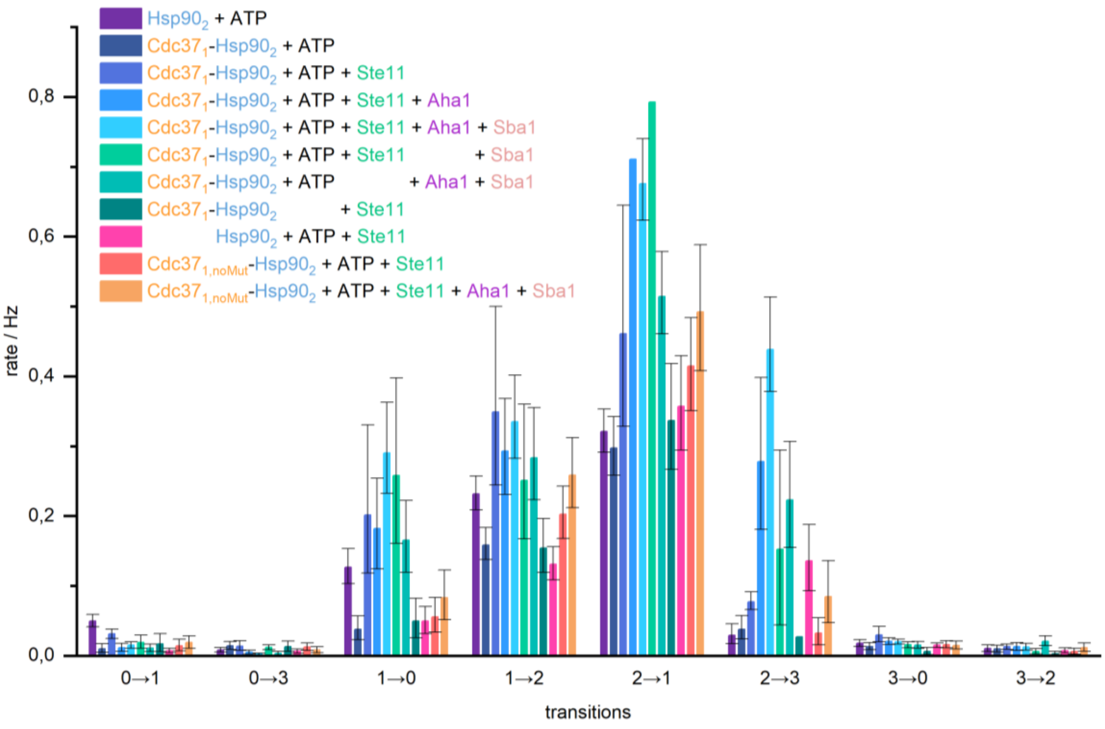


**Supplementary Fig. 4**: Mean transition rates $k_{i\text{->}j}$ for four state models of all experimental conditions from state *i* to state *j*. Error bars represent 95 % confidence intervals. Amount of traces and replicates can be found in Supplementary Table 3. Exact values of rates can be found in Supplementary Data file 1. Source data are provided as a Source Data file.

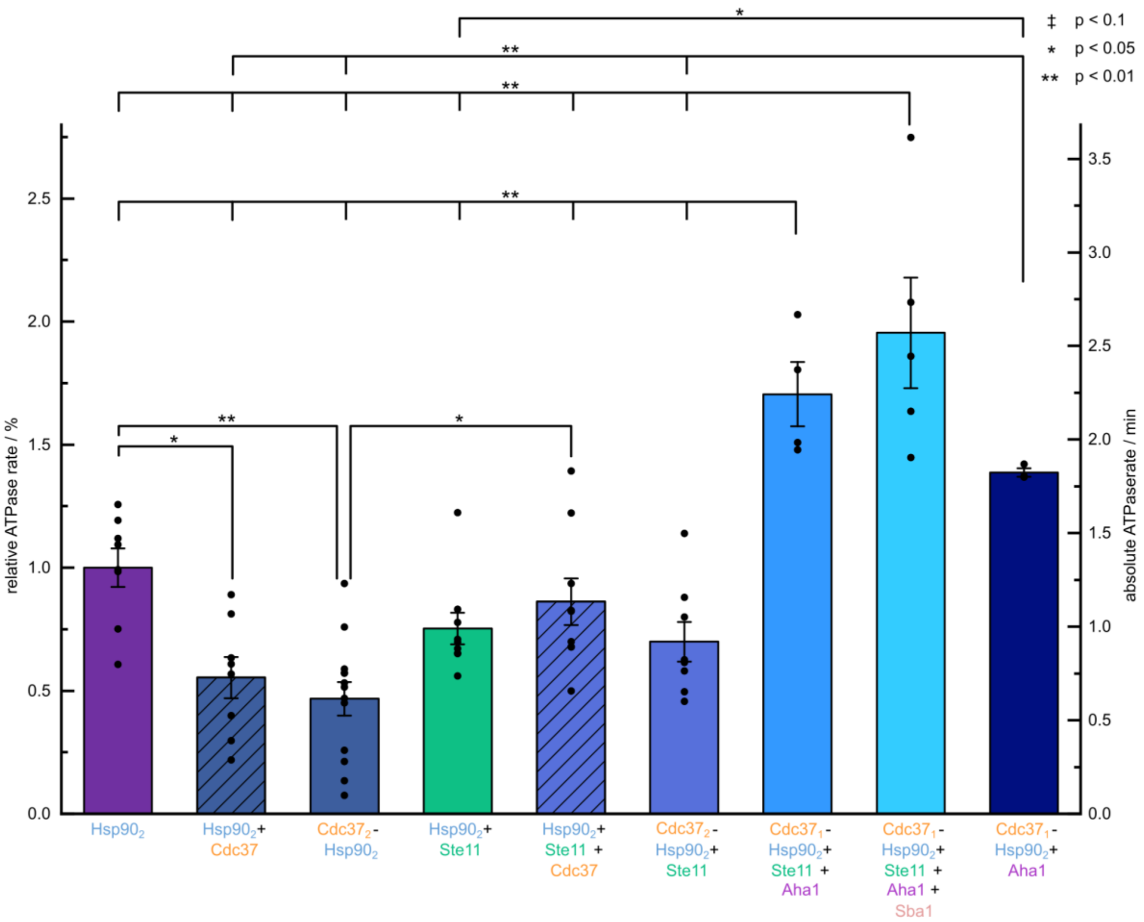


**Supplementary Fig. 5**: Relative and absolute ATPase rates ± SEM of yeast Hsp90 (2 µM) in presence and absence of cochaperones Cdc37, Aha1 and Sba1, and the client kinase Ste11.
$Hsp90_2$ (1 µM, i.e. 2 µM monomers) is a slow ATPase with 1.3 hydrolysed ATP per minute (violet). The addition of free Cdc37 (2 µM, equimolar) further decreases the ATPase rate (dark blue, striped). As the $Cdc37_2$-$Hsp90_2$-fusion shows the same behaviour, the functionality of this construct (dark blue) is proven. Ste11 (2 µM) slightly decreases Hsp90's ATPase activity (green). Additionally, Ste11 hinders Cdc37's ATPase-decreasing effect on Hsp90 of both the freely added protein (middle blue, striped) as well as the $Cdc37_2$-$Hsp90_2$-fusion (middle blue). Aha1, Cdc37, Sba1 and Ste11 together (1 µM each, lightest blue) strongly increase the ATPase to 2.6 ATP/min. This effect is not achieved by only adding Cdc37 and Aha1 (1 µM each, darkest blue). Statistical significance tested by one-way ANOVA and Tukey post hoc test (described in methods, $F(8,58) = [23.41464]$, $p = 0.05$). Source data are provided as a Source Data file.

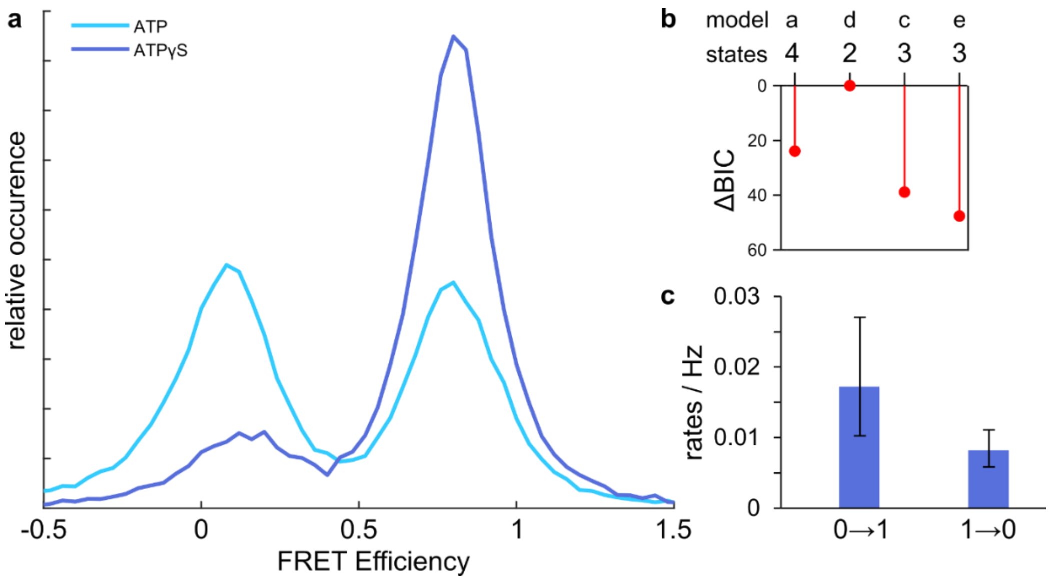


**Supplementary Fig. 6**: An energy source (e.g. ATP hydrolysis) is necessary for directed movement through a conformational cycle. **a** FRET histogram of $Cdc37_1$-$Hsp90_2$ + Ste11 + Aha1 + Sba1 in presence of ATP (light blue, 998 traces) or the almost non-hydrolysable ATP analogue ATPγS (dark blue, 435 traces). ATPγS leads to more closing of Hsp90 (83 ± 2 % closed) and few opening and closing events are observed. **b** Hidden Markov modelling in the presence of ATPγS shows that these dynamics are best described by a two state model. The same models as in Supplementary Fig. 3 were tested. State 0 represents open Hsp90, state 1 closed Hsp90. **c** Transition rates for the two state model with 95 % confidence intervals. The opening rate (1 →0) is reduced compared to the opening rate in presence of ATP (2 →1, see Fig. 3e or Supplementary Fig. 4). Source data are provided as a Source Data file.

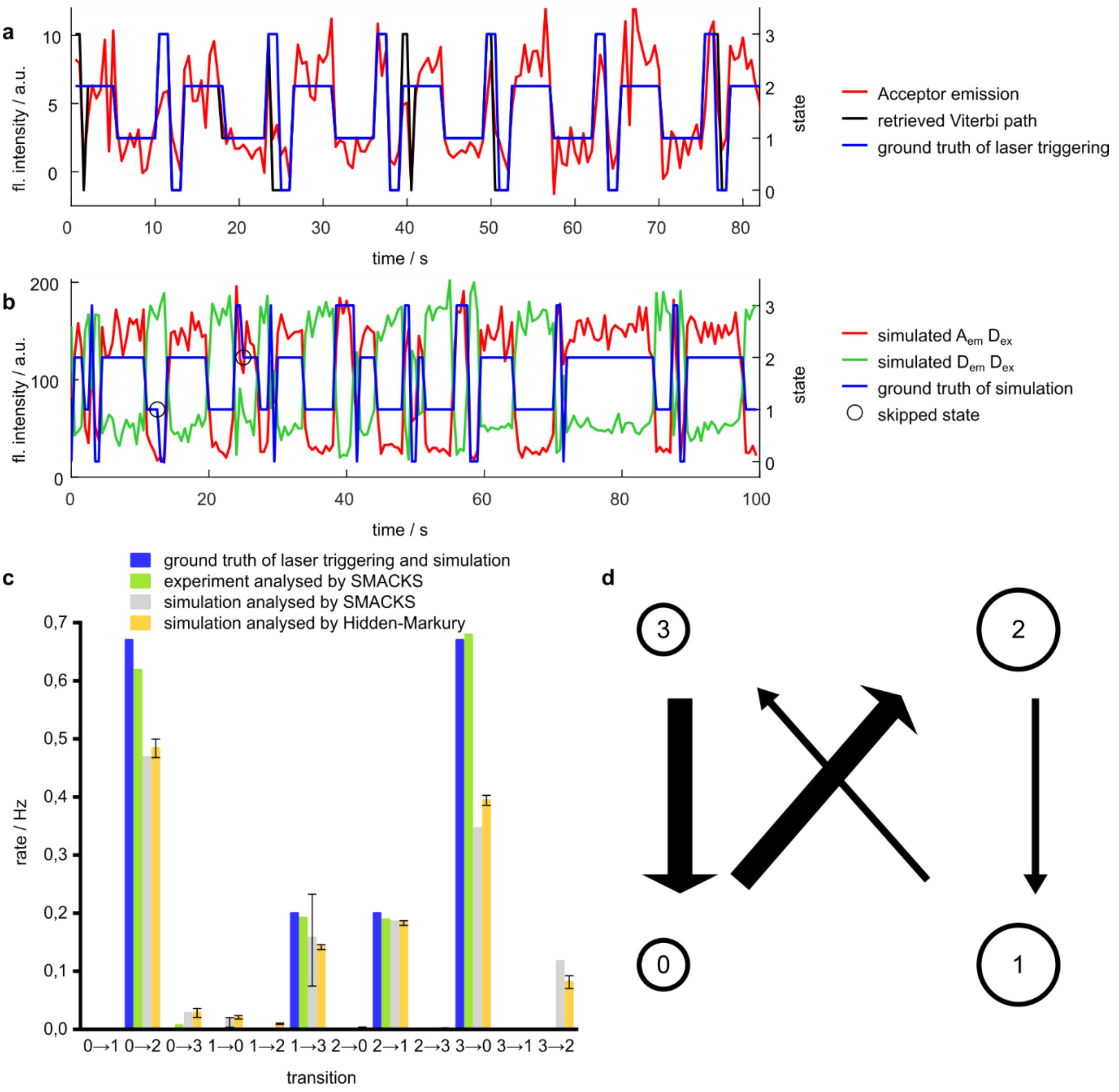


**Supplementary Fig. 7**: Deterministic (laser triggered) vs. stochastic (simulated) state sequence analysed with HMM. **a** Artificially introduced directionality by laser triggering. To achieve two distinct FRET efficiencies, a fixed low-FRET dsDNA sample is illuminated alternatingly with a green laser only (low FRET) or with a red and a green laser simultaneously (artificial high FRET). Lifetime of the states given by illumination times (1.5 vs 5 seconds). Single-molecule trace (red) of acceptor dye after donor or simultaneous excitation, respectively. Viterbi path retrieved by data analysis with SMACKS (black). **b** Simulated donor (green) and acceptor (red) signal after donor excitation with MASH-FRET. Ground truth of state allocation in blue. Due to dwell times shorter than the frame length some short-lived states (state 3 and 0) are skipped (black circles) (frame rate 2 Hz). **c** Kinetic rates retrieved from HMM analysis of laser triggered single-molecule traces (**a**, 349 traces) with SMACKS (green), from simulated traces (**b**, 200 traces) with SMACKS (grey) and Hidden-Markury (yellow). Error bars 95% Cis. **d** Model representation. Arrow thickness proportional to rates. Circle size proportional to state population. Source data are provided as a Source Data file.

**Supplementary Table 1** Results for all protein conditions from single-molecule analysis. Errors from Gaussian error propagation.

| | $Hsp90_2$<br>+ ATP | $Cdc37_1$-<br>$Hsp90_2$<br>+ ATP | $Cdc37_1$-<br>$Hsp90_2$<br>+ Ste11 | $Cdc37_1$-<br>$Hsp90_2$<br>+ ATP<br>+ Ste11 | $Cdc37_1$-<br>$Hsp90_2$<br>+ ATP<br>+ Ste11<br>+ Sba1 | $Cdc37_1$-<br>$Hsp90_2$<br>+ ATP<br>+ Ste11<br>+ Aha1<br>+ Sba1 | $Cdc37_1$-<br>$Hsp90_2$<br>+ ATP<br>+ Aha1<br>+ Sba1 | $Cdc37_1$-<br>$Hsp90_2$<br>+ ATP<br>+ Ste11<br>+ Aha1 | $Cdc37_{1, noMut}$-<br>$Hsp90_2$<br>+ ATP + Ste11<br>+ Aha1<br>+ Sba1 | $Cdc37_{1, noMut}$-<br>$Hsp90_2$<br>+ ATP<br>+ Ste11 | $Hsp90_2$<br>+ ATP<br>+ Ste11 |
|---|---|---|---|---|---|---|---|---|---|---|---|
| **closed Hsp90 ± SEM [%]** | 40.5 ± 0.4 | 48 ± 2 | 44.6 ± 0.9 | 42.8 ± 0.96 | 50.25 ± 1.01 | 46 ± 4 | 46.69 ± 0.14 | 48.7 ± 0.3 | 56.4 ± 0.6 | 53 ± 5 | 49 ± 3 |
| **$\Delta G_{mean\ rates}$ [$k_BT$]** | -0.6 | 0.7 | 0.34 | -0.45 | 0.3 | **-2.1** | -0.6 | -0.9 | -0.5 | 0.08 | -0.8 |
| **95% CI $\Delta G_{mean\ rates}$ [$k_BT$]** | [-0.8; -0.5] | [0.5; 0.8] | [0.08; 0.88] | [ -1.1; 0.04] | [-0.2; 1.0] | [-2.7; -1.7] | [-1.08; -0.45] | [-0.9; -0.9] | [-0.6; -0.4] | [-0.03; 0.15] | [-1.2; -0.6] |
| **entropy production ± error [$10^{-4}$ $k_BT$/s]** | 5 ± 13 | 4 ± 13 | 1 ± 8 | 4 ± 15 | 2 ± 10 | 26 ± 21 | 1± 11 | 5 ± 15 | 2 ± 14 | 0.4 ± 4.8 | 3 ± 9 |
| **Flux ± error [$s^{-1}$]** | 16 ± 42 | -11 ± 41 | -6 ± 47 | 18 ± 70 | -18 ± 96 | 25 ± 21 | 10 ± 40 | 11 ± 34 | 11 ± 62 | -11 ± 119 | 8 ± 23 |
| **Time per cycle ± error [min]** | 10 ± 27 | 15 ± 53 | 28 ± 225 | 9 ± 35 | 9 ± 51 | 7 ± 5 | 17 ± 68 | 15 ± 46 | 16 ± 93 | 15 ± 170 | 20 ± 59 |
| **$N_{experiments}$** | 3 | 3 | 2 | 2 | 2 | 3 | 2 | 2 | 2 | 2 | 2 |
| **$N_{sm\ traces}$** | 874 | 640 | 354 | 509 | 449 | 998 | 527 | 641 | 321 | 454 | 897 |
| **Transitions along edge [# normalized to 100]** | | | | | | | | | | | |
| **0→1** | 14.5 ± 2.1 | 4.8 ± 1.7 | 7.7 ± 1.2 | 14.2 ± 2.7 | 17.7 ± 3.8 | 21.7 ± 0.7 | 14.9 ± 0.1 | 15.2 ± 2.1 | 8.9 ± 2.5 | 8.1 ± 0.6 | 19.7 ± 4.1 |
| **0→3** | 3.2 ± 0.5 | 10.1 ± 2.2 | 6.8 ± 0.3 | 7.4 ± 2.1 | 12.2 ± 2.6 | 3.3 ± 0.4 | 4.7 ± 0.1 | 7.7 ± 1.5 | 6 ± 2 | 7.2 ± 0.2 | 8 ± 0.3 |
| **1→0** | 13.8 ± 2.3 | 6.5 ± 0.3 | 9.4 ± 0.6 | 14.4 ± 2.8 | 18.1 ± 4.9 | 17.2 ± 0.1 | 13.5 ± 1.8 | 12.9 ± 3.3 | 8.3 ± 2.4 | 8.1 ± 1.4 | 18.6 ± 5.3 |
| **1→2** | 29.4 ± 1.2 | 28.4 ± 1.6 | 29.5 ± 1.3 | 23.3 ± 1.8 | 15.4 ± 3 | 19.9 ± 1 | 22.5 ± 1.8 | 21.8 ± 1 | 29.8 ± 0.3 | 31.3 ± 0.3 | 16.7 ± 4.5 |
| **2→1** | 28.8 ± 1 | 30.1 ± 1 | 31.3 ± 0.7 | 23 ± 2.2 | 15.8 ± 1.7 | 15.3 ± 1.5 | 20.9 ± 0.2 | 19.4 ± 0.2 | 29.3 ± 0.5 | 31.4 ± 0.5 | 15.7 ± 3.4 |
| **2→3** | 2.8 ± 0.5 | 3.6 ± 0.5 | 2.5 ± 1 | 3.5 ± 1.5 | 2.8 ± 0.6 | 9.4 ± 1 | 9.1 ± 0 | 7.6 ± 0.8 | 5 ± 0 | 2.6 ± 1.4 | 5.2 ± 1.5 |
| **3→0** | 3.8 ± 0.7 | 8.2 ± 1.1 | 5 ± 0.3 | 7.7 ± 2.5 | 11.9 ± 3.9 | 7.8 ± 0.5 | 6.2 ± 2.1 | 10 ± 2.7 | 6.4 ± 1.8 | 7.1 ± 0.7 | 8.8 ± 1.1 |
| **3→2** | 2.2 ± 0.4 | 5.7 ± 0.9 | 4.3 ± 1.6 | 3. 3 ± 1.1 | 3 ± 1.8 | 5 ± 0.5 | 7.7 ± 1.8 | 5.4 ± 0.4 | 4.5 ± 0.2 | 2.7 ± 0.5 | 4.2 ± 0.4 |

**Supplementary Table 2** ΔG obtained from conditions without energy source (ATP); i.e. conditions where no directionality is possible. These measurements of Hsp90's dynamics in presence and absence of Cdc37 and Ste11 were conducted to find the lower limits of what can be definitely quantified as directional.

| | $Hsp90_2$ | $Hsp90_2$ + Ste11 | $Cdc37_1$-$Hsp90_2$ | $Cdc37_1$-$Hsp90_2$+ Ste11 |
|---|---|---|---|---|
| **ΔG [$k_BT$]** | -0.9 | 0.27 | -0.4 | 0.34 |
| **95% CI ΔG [$k_BT$]** | [-0.5;1.5] | [0.25;0.28] | [-1.0;0.7] | [0.08; 0.88] |
| **$N_{sm\text{-}traces}$** | 144 | 108 | 206 | 354 |

**Supplementary Table 3** Parameters for trace simulations.

| **Ground truth with ΔG = -3 $k_BT$** | | | | |
|---|---|---|---|---|
| **FRET E** | **$E_0$ = 0.1 ± 0.05** | **$E_1$ = 0.1 ± 0.05** | **$E_2$ = 0.8 ± 0.05** | **$E_3$ = 0.8 ± 0.05** |
| **kinetic rates [Hz]** | | $k_{01}$ = 0.02 | $k_{02}$ = 0 | $k_{03}$ = 0.002 |
| | $k_{10}$ = 0.28 | | $k_{12}$ = 0.33 | $k_{13}$ = 0 |
| | $k_{20}$ = 0 | $k_{21}$ = 0.67 | | $k_{23}$ = 0.46 |
| | $k_{30}$ = 0.025 | $k_{31}$ = 0 | $k_{32}$ = 0.01 | |
| **Ground truth with ΔG = -10 $k_BT$** | | | | |
| **FRET E** | **$E_0$ = 0.1 ± 0.05** | **$E_1$ = 0.1 ± 0.05** | **$E_2$ = 0.8 ± 0.05** | **$E_3$ = 0.8 ± 0.05** |
| **kinetic rates [Hz]** | | $k_{01}$ = 0.1 | $k_{02}$ = 0 | $k_{03}$ = 0.002 |
| | $k_{10}$ = 0.05 | | $k_{12}$ = 0.4 | $k_{13}$ = 0 |
| | $k_{20}$ = 0 | $k_{21}$ = 0.05 | | $k_{23}$ = 0.5 |
| | $k_{30}$ = 0.1 | $k_{31}$ = 0 | $k_{32}$ = 0.018 | |
| **Ground truth with ΔG = 0 $k_BT$** | | | | |
| **FRET E** | **$E_0$ = 0.1 ± 0.05** | **$E_1$ = 0.1 ± 0.05** | **$E_2$ = 0.8 ± 0.05** | **$E_3$ = 0.8 ± 0.05** |
| **kinetic rates [Hz]** | | $k_{01}$ = 0.02 | $k_{02}$ = 0 | $k_{03}$ = 0.03 |
| | $k_{10}$ = 0.28 | | $k_{12}$ = 0.33 | $k_{13}$ = 0 |
| | $k_{20}$ = 0 | $k_{21}$ = 0.67 | | $k_{23}$ = 0.46 |
| | $k_{30}$ = 0.025 | $k_{31}$ = 0 | $k_{32}$ = 0.0135 | |
| **Ground truth with ΔG = -2 $k_BT$ Hsp90 like** | | | | |
| **FRET E** | **$E_0$ = 0.1 ± 0.05** | **$E_1$ = 0.1 ± 0.05** | **$E_2$ = 0.8 ± 0.05** | **$E_3$ = 0.8 ± 0.05** |
| **kinetic rates [Hz]** | | $k_{01}$ = 0.012 | $k_{02}$ = 0 | $k_{03}$ = 0.002 |
| | $k_{10}$ = 0.29 | | $k_{12}$ = 0.31 | $k_{13}$ = 0 |
| | $k_{20}$ = 0 | $k_{21}$ = 0.69 | | $k_{23}$ = 0.4 |
| | $k_{30}$ = 0.02 | $k_{31}$ = 0 | $k_{32}$ = 0.01 | |
| **Ground truth with ΔG = -2 $k_BT$ equally distributed state populations** | | | | |
| **FRET E** | **$E_0$ = 0.1 ± 0.05** | **$E_1$ = 0.1 ± 0.05** | **$E_2$ = 0.8 ± 0.05** | **$E_3$ = 0.8 ± 0.05** |
| **kinetic rates [Hz]** | | $k_{01}$ = 0.15 | $k_{02}$ = 0 | $k_{03}$ = 0.05 |
| | $k_{10}$ = 0.1 | | $k_{12}$ = 0.5 | $k_{13}$ = 0 |
| | $k_{20}$ = 0 | $k_{21}$ = 0.3 | | $k_{23}$ = 0.15 |
| | $k_{30}$ = 0.09 | $k_{31}$ = 0 | $k_{32}$ = 0.09 | |

**Supplementary Table 4:** Parameters for laser triggering

| Acceptor fluorescence | low | low | high | high |
|---|---|---|---|---|
| **kinetic rates [Hz]** | | $k_{01}$ = 0 | $k_{02}$ = 1/1.5 | $k_{03}$ = 0 |
| | $k_{10}$ = 0 | | $k_{12}$ = 0 | $k_{13}$ = 1/5 |
| | $k_{20}$ = 0 | $k_{21}$ = 1/5 | | $k_{23}$ = 0 |
| | $k_{30}$ = 1/1.5 | $k_{31}$ = 0 | $k_{32}$ = 0 | |

## Supplementary Methods

## MASH simulation

For the simulation of single-molecule FRET traces, the program MASH-FRET was used [1]. This program uses kinetic Monte Carlo simulations to obtain the sequence of states from the given transition probabilities. The duration of the corresponding states is randomly generated from mono-exponential probability distributions, using the rate constants that characterize the interconversion between the given states. Gaussian noise is introduced to the simulated single-molecule intensity traces based on the user given FRET efficiency and its standard deviation. This is the only type of noise introduced to the traces. The simulated traces were then analyzed with SMACKS and Hidden-Markury as detailed in the main text methods and the following. The transition matrices and FRET efficiencies used as ground truth for all simulations are given in the main text methods. The results of their analyses can be found in the Supplementary Data file 1.

### Hidden Markov Analysis

Hidden Markov Analysis was performed using two different software programmes, SMACKS and Hidden Markury.

HMM by SMACKS

The functionalities of SMACKS are described by Schmid et al. [2] and in its software manual [3]. SMACKS is a maximum likelihood approach to extract kinetic rate models from noisy single-molecule data by using mathematical models for pattern recognition. Thereby, SMACKS does not rely on dwell-times but takes every experimental data point into account to optimise one global kinetic model. At the core of SMACKS' analysis lie Hidden Markov Models that establish a mathematical relation between experimental observations and their subsequent interpretation, which in turn is limited by a predefined number of states. We previously described and published a very detailed workflow for SMACKS in the Supplementary information of [4], so the following description is taken from said publication and adjusted for the data analysis done for this paper:

As a first step, donor and acceptor (FRET) fluorescence signal – both after donor excitation – from single-molecule experiments or simulations were imported. Then (in the Trace-by-Trace HMM), the number of apparent FRET states was assigned by eye. For our traces, two apparent states were chosen: one apparent high and one apparent low FRET state. Next, a Viterbi algorithm was applied to the traces to indicate these visible states. The resulting Viterbi path was checked for each trace individually. Inappropriate Viterbi paths, including the parameters causing them, were deleted. Subsequently, the mean of all correct parameters were applied to the remaining traces. Again, these traces were checked individually for correct apparent state allocation. Afterwards, in a Semi-Ensemble HMM for all traces, different state configurations with two apparent states were tested. Apparent states were denoted by different numbers (here 0 and 1), while their hidden states were indicated by doubling them (e.g., 001, 011, etc). For each model, SMACKS calculated a transition probability matrix for transitions between

different states $i$ and $j$, and a covariance matrix of the Gaussian probability densities for all states. Those probabilities $p_{ij}$ were then converted to kinetic rates $k_{ij}$ (Hz) by multiplication with the *sampling rate* used in the experiment.

$$k_{ij} = p_{ij} \cdot \text{sampling rate} \tag{1}$$

The model best representing the data was chosen after determining the BICs of each model (see Supplementary Fig. 3). SMACKS calculates 95 % confidence intervals (CIs) for the transition probabilities based on the likelihood of the obtained model.

HMM by Hidden-Markury

The Hidden-Markury software [5] is a trace analysis software based on a global optimization of one global kinetic model, which is predefined by the user. The core of the Hidden-Markov model and its optimization is based on the open python library hmmlearn (https://hmmlearn.readthedocs.io/en/latest/index.html), which uses a Viterbi algorithm, a Forward-Backward algorithm, and a Baum-Welch algorithm for a global optimization of all traces. The 2D-trace analysis was used, which uses the donor and acceptor (FRET) fluorescence signal– both after donor excitation- as input. Four-state models with two degenerate states were given to fit.

Hidden-Markury gives a mean transition rate matrix and its standard deviation (std) by bootstrapping: the model is calculated 20 times with 60 % of the traces. 95% CIs were calculated by $\mathrm{CI}_{95\%} = 1.96 \cdot \frac{\mathrm{std}}{\sqrt{20}}$.

Number of transitions

The number of transitions was calculated based on the transition matrices obtained from HMM analysis. The transitions can be calculated by multiplying the stationary state populations vector with the transition matrix. The diagonal of the resulting matrix was set to zero as staying in one's initial state does not result in a transition. For better comparability, the number of transitions was then normalized to 100. Errors for the transitions were calculated from repeats of the experiments.

**Supplementary Note 1**

Design and analysis of deterministic traces

Deterministic laser triggered single-molecule traces follow the transition matrix given in Supplementary Table 4. Even though the deterministic traces do not fulfill the requirements for a Markov process because they are not stochastic, the analysis with HMM was successful. The ground truth model described by the transition matrix would result in an undefined ΔG ($-\infty\ k_B T$). Our experiment and analysis resulted in about -165 $k_B$T, which we consider a good result considering that this way of calculation of ΔG gives a lower estimate of the free energy per cycle [6].

In addition, we simulated stochastic traces with the same transition matrix as underlying ground truth by MASH-FRET. These traces were then again analysed by SMACKS and Hidden-Markury. This resulted in a ΔG of -27 $k_B$T and -17 $k_B$T, respectively. For all transition rates, see Supplementary Fig. 7.

In the analysis of the simulated traces, the rates $k_{02}$ and $k_{30}$ are underestimated, because the short lived states 0 and 3 are skipped during simulation, when their dwell time is shorter than the frame time of 0.5 s, i.e. the frame rate 2 Hz (black circles in Supplementary Fig. 7b). This gives rise to rates $k_{01}$ and $k_{32}$.

**Supplementary Note 2**

<u>Entropy production and flux</u>

The thermodynamic force driving the four state cycle can also be called affinity $X$[7]. In the main text we use the term ΔG.

$$\Delta\mathrm{G} = -X\, k_B T \tag{2}$$

The entropy production $\langle \dot{S} \rangle$ for Markov dynamics is defined as following [6], $k_{i\to j}$ denotes the forward rate from state $i$ to $j$. The populations $P_i$ are the steady-state probabilities of finding the system in state $i$ with $\sum P_i = 1$. This gives rise to very small values for the entropy production and the flux because one could say that one molecule is distributed between four states:

$$\langle \dot{S} \rangle = \frac{1}{2}\sum_{i\neq j}\left(k_{i\to j}P_i - k_{j\to i}P_j\right)\ln\left(\frac{k_{i\to j}P_i}{k_{j\to i}P_j}\right) \tag{3}$$

For systems with a single cycle, the entropy production $\langle \dot{S} \rangle$, the affinity $X$ and the flux $J$ are connected as follows:

$$\langle \dot{S} \rangle = J\,X \tag{4}$$

The flux $J$ (net amount of transitions along one edge per unit of time), can also be calculated by the difference of forward and backward transitions [7,8].

$$J_{ij} = k_{i\to j}P_i - k_{j\to i}P_j \tag{5}$$

The inverse of the flux describes the effective time per complete cycle.

The error for entropy production, flux and effective time were calculated by Gaussian error propagation according to equation (6), (7) and (8), respectively.

$$\Delta\langle \dot{\mathrm{S}} \rangle = \sqrt{\begin{aligned}&\left(\frac{1}{2}P_0\ln\left(\frac{k_{01}P_0}{k_{10}P_1}\right)+\frac{1}{2}P_0-\frac{1}{2}\frac{P_1k_{10}}{k_{01}}\right)^2(\Delta k_{01})^2+\left(-\frac{1}{2}P_1\ln\left(\frac{k_{01}P_0}{k_{10}P_1}\right)-\frac{1}{2}\frac{P_0k_{01}}{k_{10}}+\frac{1}{2}P_1\right)^2(\Delta k_{10})^2\\&+\left(\frac{1}{2}P_1\ln\left(\frac{k_{12}P_1}{k_{21}P_2}\right)+\frac{1}{2}P_1-\frac{1}{2}\frac{P_2k_{21}}{k_{12}}\right)^2(\Delta k_{12})^2+\left(-\frac{1}{2}P_2\ln\left(\frac{k_{12}P_1}{k_{21}P_2}\right)-\frac{1}{2}\frac{P_1k_{12}}{k_{21}}+\frac{1}{2}P_2\right)^2(\Delta k_{21})^2\\&+\left(\frac{1}{2}P_2\ln\left(\frac{k_{23}P_2}{k_{32}P_3}\right)+\frac{1}{2}P_2-\frac{1}{2}\frac{P_3k_{32}}{k_{23}}\right)^2(\Delta k_{23})^2+\left(-\frac{1}{2}P_3\ln\left(\frac{k_{23}P_2}{k_{32}P_3}\right)-\frac{1}{2}\frac{P_2k_{23}}{k_{32}}+\frac{1}{2}P_3\right)^2(\Delta k_{32})^2\\&+\left(\frac{1}{2}P_3\ln\left(\frac{k_{30}P_3}{k_{03}P_0}\right)+\frac{1}{2}P_3-\frac{1}{2}\frac{P_0k_{03}}{k_{30}}\right)^2(\Delta k_{30})^2+\left(-\frac{1}{2}P_0\ln\left(\frac{k_{30}P_3}{k_{03}P_0}\right)-\frac{1}{2}\frac{P_3k_{30}}{k_{03}}+\frac{1}{2}P_0\right)^2(\Delta k_{03})^2\end{aligned}} \tag{6}$$

$$\Delta\mathrm{J} = \sqrt{\left(\frac{-2}{\Delta\mathrm{G}}\right)^2\left(\Delta\langle\dot{\mathrm{S}}\rangle\right)^2+\left(\frac{2\langle\dot{S}\rangle}{\Delta\mathrm{G}^2}\right)^2(\Delta\Delta\mathrm{G})^2} \tag{7}$$

$$\Delta\mathrm{effTime} = \sqrt{\left(\frac{-1}{120\langle\dot{S}\rangle}\right)^2(\Delta\Delta\mathrm{G})^2+\left(\frac{\Delta\mathrm{G}}{120\langle\dot{S}\rangle^2}\right)^2\left(\Delta\langle\dot{\mathrm{S}}\rangle\right)^2}\ \text{in min} \tag{8}$$

Idle Hsp90 has an ATPase rate of ~ 1 ATP/min, but its energy is not used for a directional movement through the cycle. Only when Cdc37, Sba1, Aha1 and Ste11 are present we can observe a ΔG of -2.1 $k_BT$ (condition $Cdc37_1$-$Hsp90_2$ + ATP + Ste11 + Aha1 + Sba1). One ATP gives ~-20 $k_BT$ of energy [9], which means that in our system with a directional cycle, only about 10% of the total energy of ATP hydrolysis is coupled into this cycle. When calculating the reciprocal value of the flux of the system, we get the

effective time needed for one successful cycle. In the condition where we observe directionality ($Cdc37_1$-$Hsp90_2$ + ATP + Ste11 + Aha1 + Sba1), this effective time is (7±5) min. 7 min per cycle and an efficiency of about 10% results in about 40 s for the ATP hydrolysis time, i.e. 1.5 ATP/min. This agrees very well with the measured ATPase rate of 2 ATP/min under these conditions.

**Supplementary References**


1. Börner, R. *et al.* Simulations of camera-based single-molecule fluorescence experiments. *PloS one* **13,** e0195277; 10.1371/journal.pone.0195277 (2018).
2. Schmid, S., Götz, M. & Hugel, T. Single-Molecule Analysis beyond Dwell Times: Demonstration and Assessment in and out of Equilibrium. *Biophysical journal* **111,** 1375–1384; 10.1016/j.bpj.2016.08.023 (2016).
3. Schmid, S. & Götz, M. *SMACKS. Single Molecule Analysis of Complex Kinetic Sequences* (2016).
4. Götz, M. *et al.* A blind benchmark of analysis tools to infer kinetic rate constants from single-molecule FRET trajectories. *Nature communications* **13,** 5402; 10.1038/s41467-022-33023-3 (2022).
5. Gebhardt, C. *Hidden Markury* (GitHub, 2021).
6. Godec, A. & Makarov, D. E. Challenges in Inferring the Directionality of Active Molecular Processes from Single-Molecule Fluorescence Resonance Energy Transfer Trajectories. *The journal of physical chemistry letters* **14,** 49–56; 10.1021/acs.jpclett.2c03244 (2023).
7. Hill, T. L. *Free Energy Transduction and Biochemical Cycle Kinetics* (Springer New York, New York, NY, 1989).
8. Seifert, U. Stochastic thermodynamics, fluctuation theorems and molecular machines. *Reports on progress in physics. Physical Society (Great Britain)* **75,** 126001; 10.1088/0034-4885/75/12/126001 (2012).
9. Phillips, R. C., George, P. & Rutman, R. J. Thermodynamic Data for the Hydrolysis of Adenosine Triphosphate as a Function of pH, Mg2+ Ion Concentration, and Ionic Strength. *Journal of Biological Chemistry* **244,** 3330–3342; 10.1016/S0021-9258(18)93131-5 (1969).
