# Supplementary material for: Cochaperones convey the energy of ATP hydrolysis for directional action of Hsp90
Source: Nat Commun. 2024 Jan 17;15:569. doi: 10.1038/s41467-024-44847-6 (PMC10794413; doi:10.1038/s41467-024-44847-6)
Supplement: Supplementary file 3 — Description of Additional Supplementary Files [file 41467_2024_44847_MOESM3_ESM.pdf]

Description of Additional Supplementary Files

File name: Supplementary Code 1

Description: Code for the laser triggering and for single-molecule video analysis.
