# Supplementary material for: Cochaperones convey the energy of ATP hydrolysis for directional action of Hsp90
Source: Nat Commun. 2024 Jan 17;15:569. doi: 10.1038/s41467-024-44847-6 (PMC10794413; doi:10.1038/s41467-024-44847-6)
Supplement: Supplementary file 4 — Supplementary Code 1 [file 41467_2024_44847_MOESM4_ESM.zip › Read Me.pdf]

File name: Supplementary Code 1

Description: Labview:

Laser triggering:

Lasers were triggered by using an in-house LabVIEW program. The Camera, shutters and lasers need to be connected to the Digital-I/O-device NI PCIe-6535 (National Instruments).

To start the program, open MAIN_Trigger.vi in LabVIEW 2019 (National Instruments, quick typical install time on a regular PC).

Load the trigger file (e.g. g_r_200+50_directionality_red.txt) by selecting it in its folder and clicking "Laden". Now the trigger sequence can be started using the START button.

Igor: single molecule video analysis

Analysis pipeline:

1. Selection of single-molecule time traces

iniTIRF.ipf is an Igor Pro (Version 6.37.2, WaveMetrics, quick typical install time on a regular PC) based in-house script and started by opening it in Igor Pro and compiling it.

Image registration with fluorescent beads (TetraSpeck microspheres, 0.2 µm, Invitrogen) preceded the experiments to align the green and the red channel.

The single-molecule data was recorded as movies and donor and acceptor fluorescence signal was saved in a master and a slave TIFF (tag image file format) stack, respectively. These tiff stacks were then imported to the "CR 2f alex: ex g/r: det g+r/r" setup using the button "Load Film" available on the GUI. After selecting the red_green (i.e FRET) channel on the "apply to channel" tab, the button "find traces" opens the single-molecule time traces.

The program identifies the positions of single molecules by searching for the brightest spots in five consecutive frames (this can be specified in the "sum frames" tab) of the respective detection channel. With the buttons "+S" (forward) and "-S" (backwards), the next/previous set of time traces can be loaded into the timeline window.

In the timeline window, traces are selected with the cursor by simultaneously pressing "ctrl" and the left mouse button, then using the "save" button. When selecting the first trace, another window opens ("pFrameTime"), in which the correct frame time needs to be specified (in our case: 200 ms exposure, 50 ms read out time, 200 ms exposure, 50 ms read out time).

This procedure is repeated for all recorded videos.

After going through all recorded videos, the saved traces can be displayed by choosing the option "show saved traces" in the "smFRET new" tab. Pressing the button "use it!", another window opens displaying the first of your single-molecule traces. Here, the Donor (green trace) or Acceptor only (black trace) as well as the FRET part (red trace) of the traces can be selected. The blue signal shows the sum of acceptor

and donor signal and can be hidden by clicking on the “SUM” button. To select the ranges, simultaneously press “ctrl” and click and hold the left mouse button on one end of the Donor/Acceptor only signal, then go to the other end of the Donor/Acceptor signal and release the mouse button. Press the “SvE Only” button. Repeat the procedure for the FRET range and press “SvE range”. The program automatically directs you to the next trace. Repeat the procedure until you have selected ranges for all your traces. In case you have accidentally chosen traces that do not show good FRET or acceptor/donor only ranges, you can skip them by using “”. Criteria for good traces are flat intensity plateaus, a single bleaching step for all excitation colors, and anti-correlated behavior in the appropriate detection channels.

Data was corrected as described in Hellenkamp, B., Schmid, S., Doroshenko, O. *et al*. Precision and accuracy of single-molecule FRET measurements—a multi-laboratory benchmark study. *Nat Methods* **15**, 669–676 (2018). https://doi.org/10.1038/s41592-018-0085-0

2. Kinetic analysis

The selected, corrected FRET ranges were then loaded into the different analysis tools (SMACKS, MASH-FRET, Hidden-Markury) to infer kinetic rate constants. We followed the manuals provided by the analysis tools.
