# Supplementary material for: Cochaperones convey the energy of ATP hydrolysis for directional action of Hsp90
Source: Nat Commun. 2024 Jan 17;15:569. doi: 10.1038/s41467-024-44847-6 (PMC10794413; doi:10.1038/s41467-024-44847-6)
Supplement: Supplementary file 5 — Reporting Summary [file 41467_2024_44847_MOESM5_ESM.pdf]

nature portfolio

Corresponding author(s): Thorsten Hugel

Last updated by author(s): Dec 18, 2023

Reporting Summary

Nature Portfolio wishes to improve the reproducibility of the work that we publish. This form provides structure for consistency and transparency in reporting. For further information on Nature Portfolio policies, see our Editorial Policies and the Editorial Policy Checklist.

Statistics

For all statistical analyses, confirm that the following items are present in the figure legend, table legend, main text, or Methods section.

n/a	Confirmed	
☐	☒	The exact sample size (*n*) for each experimental group/condition, given as a discrete number and unit of measurement
☒	☐	A statement on whether measurements were taken from distinct samples or whether the same sample was measured repeatedly
☐	☒	The statistical test(s) used AND whether they are one- or two-sided<br>*Only common tests should be described solely by name; describe more complex techniques in the Methods section.*
☒	☐	A description of all covariates tested
☐	☒	A description of any assumptions or corrections, such as tests of normality and adjustment for multiple comparisons
☐	☒	A full description of the statistical parameters including central tendency (e.g. means) or other basic estimates (e.g. regression coefficient) AND variation (e.g. standard deviation) or associated estimates of uncertainty (e.g. confidence intervals)
☐	☒	For null hypothesis testing, the test statistic (e.g. *F*, *t*, *r*) with confidence intervals, effect sizes, degrees of freedom and *P* value noted<br>*Give P values as exact values whenever suitable.*
☒	☐	For Bayesian analysis, information on the choice of priors and Markov chain Monte Carlo settings
☒	☐	For hierarchical and complex designs, identification of the appropriate level for tests and full reporting of outcomes
☒	☐	Estimates of effect sizes (e.g. Cohen's *d*, Pearson's *r*), indicating how they were calculated

Our web collection on statistics for biologists contains articles on many of the points above.

Software and code

Policy information about availability of computer code

Data collection	Laser tigger software given in Supplementary Information run in LabView 2019 (Version 19.0f2), commercial Andor SOLIS for Imaging (Version 4.30.30034.0)
Data analysis	All software tools are available: iniTIRF given in Supplementary Information run in Igor Pro 6.37 (WaveMetrics, Version 6.3.7.2), SMACKS v1.4 (https://github.com/sciSonja/SMACKS/), MASH-FRET v.1.3.2 (https://github.com/RNA-FRETools/MASH-FRET), Hidden-Markury v 1.0.1 (https://github.com/ChristianGebhardt/Hidden-Markury); commercial OriginPro 2021 (Version 9.8.0.200)

For manuscripts utilizing custom algorithms or software that are central to the research but not yet described in published literature, software must be made available to editors and reviewers. We strongly encourage code deposition in a community repository (e.g. GitHub). See the Nature Portfolio guidelines for submitting code & software for further information.

Data

Policy information about availability of data

All manuscripts must include a data availability statement. This statement should provide the following information, where applicable:

- Accession codes, unique identifiers, or web links for publicly available datasets
- A description of any restrictions on data availability
- For clinical datasets or third party data, please ensure that the statement adheres to our policy

All data generated or analysed during this study are included in this published article in the Supplementary Information and Source Data. Supplementary figures, notes, and methods are provided in the Supplementary Information file. Source data for all figures are provided as Source data file with this paper.

Research involving human participants, their data, or biological material

Policy information about studies with human participants or human data. See also policy information about sex, gender (identity/presentation), and sexual orientation and race, ethnicity and racism.

Reporting on sex and gender	not applicable
Reporting on race, ethnicity, or other socially relevant groupings	not applicable
Population characteristics	not applicable
Recruitment	not applicable
Ethics oversight	not applicable

Note that full information on the approval of the study protocol must also be provided in the manuscript.

Field-specific reporting

Please select the one below that is the best fit for your research. If you are not sure, read the appropriate sections before making your selection.

☒ Life sciences ☐ Behavioural & social sciences ☐ Ecological, evolutionary & environmental sciences

For a reference copy of the document with all sections, see nature.com/documents/nr-reporting-summary-flat.pdf

Life sciences study design

All studies must disclose on these points even when the disclosure is negative.

Sample size	We have analysed more than 321 proteins for every condition (more than 6600 in total) and at least two independent protein batches. The number of proteins was chosen such that the confidence intervals and standard deviations for the calculated quantities are small. This ensured that the behaviour observed was statistically significant. Selection criteria are clearly specified.
Data exclusions	No data was excluded.
Replication	Independent replicates were performed for every experiment. The exact numbers are given in the Source Data and supplementary Information. All attempts of replication were successfull and are included in this study.
Randomization	Randomization was not relevant because no groups were allocated in our study.
Blinding	Blinding was not relevant because no groups were allocated in our study.

Reporting for specific materials, systems and methods

We require information from authors about some types of materials, experimental systems and methods used in many studies. Here, indicate whether each material, system or method listed is relevant to your study. If you are not sure if a list item applies to your research, read the appropriate section before selecting a response.

Materials & experimental systems

n/a	Involved in the study
☒	☐ Antibodies
☒	☐ Eukaryotic cell lines
☒	☐ Palaeontology and archaeology
☒	☐ Animals and other organisms
☒	☐ Clinical data
☒	☐ Dual use research of concern
☒	☐ Plants

Methods

n/a	Involved in the study
☒	☐ ChIP-seq
☒	☐ Flow cytometry
☒	☐ MRI-based neuroimaging
